# Cepacia Syndrome in a Non-Cystic Fibrosis Patient

**DOI:** 10.1155/2015/537627

**Published:** 2015-08-18

**Authors:** Naomi Hauser, Jose Orsini

**Affiliations:** Department of Medicine, New York University School of Medicine, Woodhull Medical and Mental Health Center, Brooklyn, NY 11206, USA

## Abstract

*Burkholderia* (formerly *Pseudomonas*) *cepacia* complex is a known serious threat to patients with cystic fibrosis, in whom it has the potential to cause the fatal combination of necrotizing pneumonia, worsening respiratory failure, and bacteremia, known as Cepacia syndrome. The potential for this pathogen to infect non-cystic fibrosis patients is limited and its epidemiology is poorly understood. Previously reported cases of severe *Burkholderia cepacia* complex lung infection in immunocompetent hosts include pneumonia, bronchiectasis, pyopneumothorax, and cavitary lesions. We present a case of a 64-year-old man with *Streptococcus pneumoniae* community-acquired pneumonia whose hospital course was complicated by developing cavitary lung lesions, bacteremia, and acute respiratory distress syndrome. Repeated tracheal aspirate and blood cultures grew *Burkholderia cepacia*. Our case appears to be the first report of Cepacia syndrome in a patient without cystic fibrosis. This report raises concern regarding the potential severity of pulmonary *Burkholderia cepacia* complex infection and the need to broaden clinicians' suspicion for Cepacia syndrome. A framework to help diagnose and treat infected non-cystic fibrosis individuals may be useful.

## 1. Introduction

*Burkholderia* (formerly* Pseudomonas*)* cepacia* complex (Bcc) has been a recognized serious threat to patients with cystic fibrosis (CF) since it emerged as a major cause of severe pulmonary infection in this population in the 1980s [1–3]. Bcc is known to colonize the lungs of CF patients, occasionally causing chronic pulmonary infection [3–8]. The greatest concern of pulmonary Bcc infection is its potential to cause Cepacia syndrome, a fatal combination of necrotizing pneumonia, rapid respiratory decline, and bacteremia [3, 5, 8–11]. The potential of Bcc to infect non-CF patients is limited and sporadic, and its epidemiology is poorly understood [1, 8]. Large hospital outbreaks are infrequent [2]. Infection in the non-CF population is most commonly nosocomial and due to direct delivery via some clear point source, with resolution of illness upon removing the exposure [8, 12, 13]. Cases of severe Bcc lung infection in immunocompetent hosts have been reported in the literature and include pneumonia, bronchiectasis, pyopneumothorax, and cavitary lesions [7, 14–18].  Table 1 summarizes cases reported in the literature of necrotizing pneumonia due to Bcc in non-CF individuals. To the best of our knowledge, however, this is one very rare case of classic Cepacia syndrome in a non-CF individual and the only case of Cepacia syndrome due to nosocomial Bcc infection reported in the literature.

## 2. Case Report

A 64-year-old man presented to the emergency department (ED) complaining of right pleuritic pain, productive cough, and generalized malaise for 4 days. His past medical history was significant for systemic arterial hypertension and type 2 diabetes mellitus. Vital signs on arrival to the ED were: blood pressure of 120/75 mmHg, heart rate of 114 beats/minute, respiratory rate of 22 breaths/minute, and temperature of 96.9°F. His oxygen saturation by pulse oximetry was 89% while breathing ambient air. Coarse rales were heard on auscultation over the right lung field. The rest of the physical exam was noncontributory.

**Figure 1:**
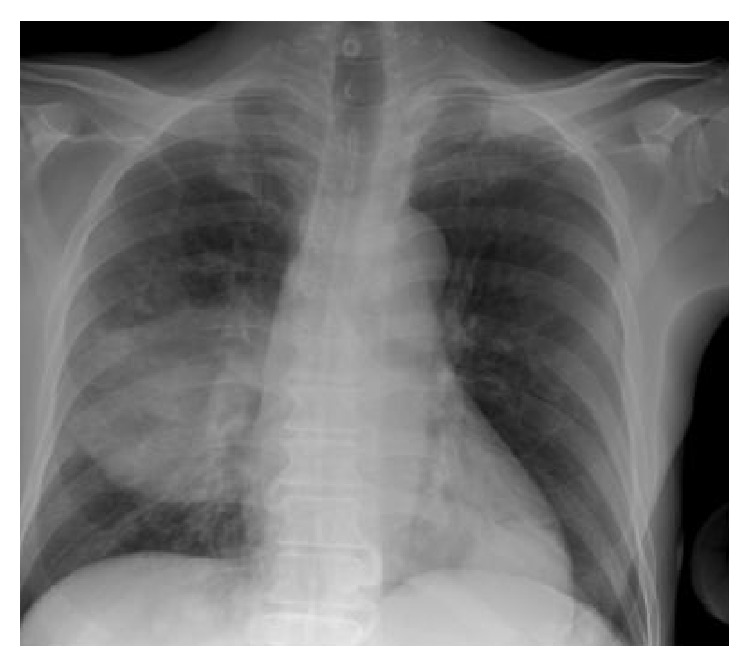


**Figure 2:**
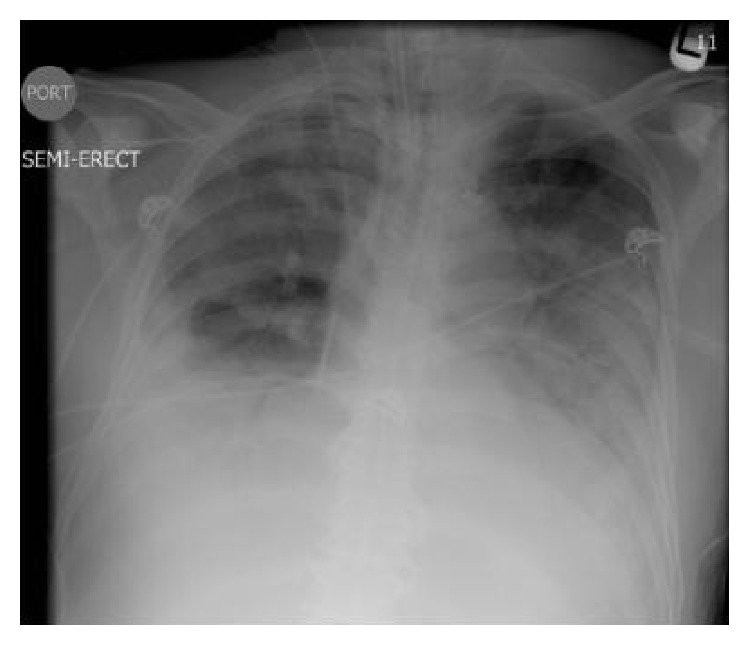


**Figure 3:**
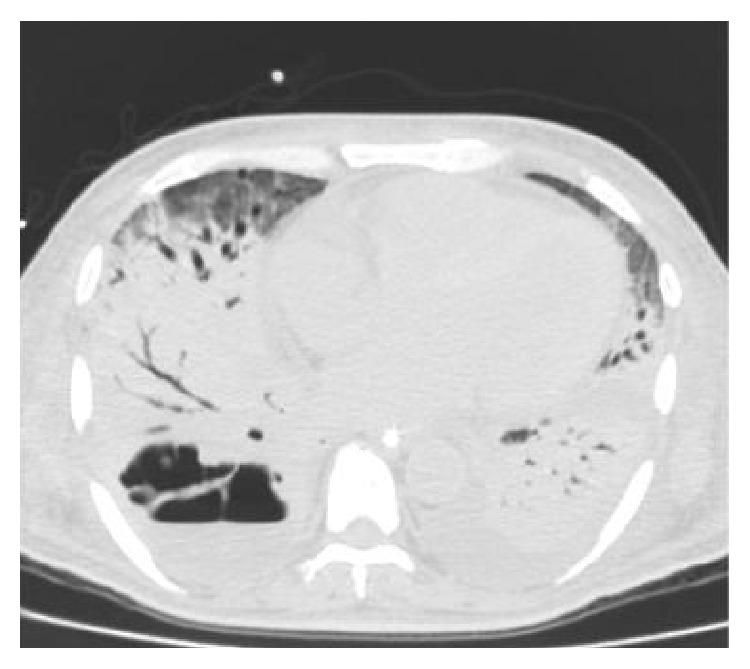


Remarkable laboratory findings included a sodium level of 126 mmol/L (135–145), a bicarbonate level of 7 mmol/L (24–31), a creatinine level of 1.9 mg/L (0.8–2.0), and a glucose of 687 mg/dL (65–115). Arterial blood gas (ABG) showed paO_2_ of 59 mmHg (80–100) on room air. Chest X-ray (CXR) demonstrated right middle lobe consolidation (Figure 1). The patient was admitted to the intensive care unit (ICU) with diagnoses of diabetic ketoacidosis (DKA) and acute hypoxemic respiratory failure secondary to community-acquired pneumonia. Empiric intravenous antimicrobial therapy was initiated comprising ceftriaxone (2 grams every 12 hours) and azithromycin (500 milligrams daily). Blood cultures obtained on admission grew* Streptococcus pneumoniae*, and azithromycin was discontinued. The patient's medical condition improved and he was transferred to a medical ward on hospital day 2. His clinical course was complicated by worsening tachypnea, leukocytosis of 21,700/mm^3^ (4,500–10,400), and hypoxemia. ABG while receiving 40% venturi mask showed paO_2_ of 46 mmHg. Repeat CXR showed worsening bilateral infiltrates (Figure 2). He was intubated, placed on mechanical ventilation, and readmitted to the ICU with the diagnosis of acute respiratory distress syndrome (ARDS). Ceftriaxone was replaced with intravenous vancomycin (1 gram every 12 hours) and cefepime (2 grams every 12 hours). A chest computed tomography (CT) with contrast showed a large consolidation with air bronchograms involving the superior segment of the right lower lobe, with a cavitary-like lesion with air fluid levels in the posterior aspect of the right hemithorax, suggestive of empyema, necrotizing pneumonia, or abscess (Figure 3). His ICU course was further complicated by refractory shock and multiorgan dysfunction syndrome. Blood and bronchoalveolar lavage (BAL) cultures obtained in the ICU grew* Burkholderia cepacia* complex. Antimicrobial therapy was substituted with doripenem 250 milligrams intravenously every 8 hours, adjusted to creatinine clearance. Despite all medical therapy, the patient expired on the 30th day of ICU admission.

## 3. Literature Review

A review of the literature was undertaken to compare our case to other reported cases of necrotizing pneumonia due to Bcc in non-CF patients and the results are listed in Table 1. PubMed search terms “Cepacia syndrome AND immunocompetent,” “Cepacia syndrome AND non-cystic fibrosis,” and “Cepacia syndrome AND without cystic fibrosis” yielded no results. A PubMed search using search terms “*Burkholderia cepacia* AND necrotizing pneumonia AND immunocompetent” yielded only one report of cases of necrotizing pneumonia due to Bcc in non-CF patients [18]. A Google Scholar search using the same search terms also yielded one case report [16]. Belchis et al. in 2000 described 3 cases of community-acquired necrotizing pneumonia caused by Bcc in men and women in their 40s, one of which also had bacteremia [18]. All 3 cases resulted in rapid clinical decline and death. Suresh et al. in 2013 described an additional case of community-acquired necrotizing pneumonia with lung cavitation in a 63-year-old man [16]. It is unclear whether or not this man was also bacteremic. He did not experience the rapid decline that defines Cepacia syndrome, however, and complete recovery was observed within 6 weeks.

When compared to our case, these previous reports of necrotizing pneumonia caused by Bcc infection in non-CF individuals were community-acquired with varying bacteremia status. Three of the cases experienced rapid decline and death. Only one of the four cases meets all of necrotizing pneumonia, bacteremia, and rapid respiratory decline which are necessary qualifications for Cepacia syndrome. Our case of a hospitalized patient with necrotizing pneumonia and bacteremia due to Bcc infection appears to be a very rare case of Cepacia syndrome in a non-CF individual and maybe the first case due to nosocomial infection.

## 4. Discussion

While Bcc is a known threat to patients with CF, its potential to infect and harm non-CF individuals is less well established. Infection in the non-CF population is most commonly nosocomial via contaminated hospital equipment, but horizontal transmission from CF patients has been reported [7, 8, 12, 13, 19]. Bcc bacteremia in non-CF patients is most often related to direct bloodstream access via contaminated peripheral or central intravenous catheters, with resolution of symptoms after removal of the contaminated equipment [13]. Pulmonary infection in non-CF patients is similarly nosocomial and nearly always related to mechanical ventilation and contaminated water [12]. We suspect that our patient most likely acquired the infection via respiratory equipment, as he received nebulizer therapy, noninvasive ventilation, and ultimately invasive ventilation over the course of his hospital stay. Other reported sources within hospitals described in the literature include disinfectants, antiseptics, topical anesthetics, respiratory therapy equipment, mouthwash, and lotions [20–22]. Horizontal transmission between CF and non-CF patients is thought to occur in some hospital situations when infection control guidelines are unclear or inadequately followed [8, 19]. Ledson et al. described a rare case of social Bcc transmission from colonized children with CF to their healthy, non-CF mother outside of the hospital, causing a chronic bronchiectasis [7]. In many cases, however, the source of infection remains unknown [8, 19, 21].

Bressler et al. found that significant risk factors for Bcc bacteremia in non-CF patients include having had at least 2 bronchoscopic procedures, having an indwelling venous catheter, renal failure requiring dialysis, tracheostomy, and recent abdominal surgery [8]. The patient described in this report had two of these hypothesized risk factors: a central venous catheter and renal replacement therapy. Despite their identification of several physical risk factors, causality was difficult to establish. Cases of Cepacia syndrome with varying species of Bcc have been reported in CF patients known to be chronic respiratory carriers [4, 9]. Cepacia syndrome is nearly always fatal, with only very few cases reported of successful treatment, all in CF patients [3, 5, 11]. Cases of severe Bcc lung infection other than Cepacia syndrome have been reported in immunocompetent hosts, most of which survived with appropriate antimicrobial therapy [7, 14–16]. To our knowledge, however, this is the first report of Cepacia syndrome in a non-CF individual.

Historically, there has been argument over the role of Bcc as a disease-causing agent or merely as a marker of underlying lung disease [10]. For this reason, controversy remains over precautionary infection control measures once Bcc infection in non-CF patients has been identified [10, 19, 21]. Our case raises concern regarding the potential lethality of pulmonary Bcc infection in the non-CF host and the need for a framework to help guide early diagnosis and treatment of infected individuals.

## Figures and Tables

**Table 1:** Summary of case reports of necrotizing pneumonia due to *Burkholderia cepacia* complex species in non-cystic fibrosis patients.

Author, year	Age, gender	Exposure	Bacteremia	Rapid respiratory decline	Outcome
Belchis et al., 2000 [18]	44, M	Community	Yes	Yes	Death
Belchis et al., 2000 [18]	40, F	Community	No	Yes	Death
Belchis et al., 2000 [18]	43, F	Community	No	Yes	Death
Suresh et al., 2013 [16]	63, M	Community	Unclear	No	Survival

## References

[1] Holmes A., Govan J., Goldstein R. (1998). Agricultural use of *Burkholderia* (*Pseudomonas*) *cepacia*: a threat to human health?. *Emerging Infectious Diseases*.

[2] Reik R., Spilker T., LiPuma J. J. (2005). Distribution of *Burkholderia cepacia* complex species among isolates recovered from persons with or without cystic fibrosis. *Journal of Clinical Microbiology*.

[3] Weidmann A., Webb A. K., Dodd M. E., Jones A. M. (2008). Successful treatment of cepacia syndrome with combination nebulised and intravenous antibiotic therapy. *Journal of Cystic Fibrosis*.

[4] Shafiq I., Carroll M. P., Nightingale J. A., Daniels T. V. W. (2011). Cepacia syndrome in a cystic fibrosis patient colonised with *Burkholderia multivorans*. *BMJ Case Reports*.

[5] Gilchrist F. J., Webb A. K., Bright-Thomas R. J., Jones A. M. (2012). Successful treatment of cepacia syndrome with a combination of intravenous cyclosporin, antibiotics and oral corticosteroids. *Journal of Cystic Fibrosis*.

[6] Blackburn L., Brownlee K., Conway S., Denton M. (2004). ‘Cepacia syndrome’ with *Burkholderia multivorans*, 9 years after initial colonization. *Journal of Cystic Fibrosis*.

[7] Ledson M. J., Gallagher M. J., Walshaw M. J. (1998). Chronic *Burkholderia cepacia* bronchiectasis in a non-cystic fibrosis individual. *Thorax*.

[8] Bressler A. M., Kaye K. S., LiPuma J. J. (2007). Risk factors for *Burkholderia cepacia* complex bacteremia among intensive care unit patients without cystic fibrosis: a case-control study. *Infection Control and Hospital Epidemiology*.

[9] Zahariadis G., Levy M. H., Burns J. L. (2003). Cepacia-like syndrome caused by *Burkholderia multivorans*. *Canadian Journal of Infectious Diseases*.

[10] Govan J. R. W., Hughes J. E., Vandamme P. (1996). *Burkholderia cepacia*: medical, taxonomic and ecological issues. *Journal of Medical Microbiology*.

[11] Grimwood K., Kidd T. J., Tweed M. (2009). Successful treatment of cepacia syndrome. *Journal of Cystic Fibrosis*.

[12] Conly J. M., Klass L., Larson L., Kennedy J., Low D. E., Harding G. K. (1986). *Pseudomonas cepacia* colonization and infection in intensive care units. *Canadian Medical Association Journal*.

[13] Meyer G. W. (1973). *Pseudomonas cepacia* septicemia associated with intravenous therapy. *California Medicine*.

[14] Waterer G. W., Jones C. B., Wunderink R. G. (1999). Bacteremic community-acquired pneumonia in an immunocompetent adult due to *Burkholderia cepacia*. *Chest*.

[15] Karanth S. S., Regunath H., Chawla K., Prabhu M. (2012). A rare case of community acquired *Burkholderia cepacia* infection presenting as pyopneumothorax in an immunocompetent individual. *Asian Pacific Journal of Tropical Biomedicine*.

[16] Suresh G., Prakasha S. R., Giridhar B. H., Prakash K. S. (2013). Cavity in the lung: a rare case of *Burkholderia cepacia* infection. *Nitte University Journal of Health Science*.

[17] Pujol M., Corbella X., Carratala J., Gudiol F. (1992). Community-acquired bacteremic *Pseudomonas cepacia* pneumonia in an immunocompetent host. *Clinical Infectious Diseases*.

[18] Belchis D. A., Simpson E., Colby T. (2000). Histopathologic features of *Burkholderia cepacia* pneumonia in patients without cystic fibrosis. *Modern Pathology*.

[19] Holmes A., Nolan R., Taylor R. (1999). An epidemic of *Burkholderia cepacia* transmitted between patients with and without cystic fibrosis. *The Journal of Infectious Diseases*.

[20] Jones A. M., Dodd M. E., Webb A. K. (2001). *Burkholderia cepacia*: current clinical issues, environmental controversies and ethical dilemmas. *European Respiratory Journal*.

[21] Siddiqui A. H., Mulligan M. E., Mahenthiralingam E. (2001). An episodic outbreak of genetically related *Burkholderia cepacia* among non-cystic fibrosis patients at a university hospital. *Infection Control and Hospital Epidemiology*.

[22] Zurita J., Mejia L., Zapata S. (2014). Healthcare-associated respiratory tract infection and colonization in an intensive care unit caused by *Burkholderia cepacia* isolated in mouthwash. *International Journal of Infectious Diseases*.

